# Healthy eating index patterns in adults by sex and age predict cardiometabolic risk factors in a cross-sectional study

**DOI:** 10.1186/s40795-021-00432-4

**Published:** 2021-06-22

**Authors:** Virginia M. Artegoitia, Sridevi Krishnan, Ellen L. Bonnel, Charles B. Stephensen, Nancy L. Keim, John W. Newman

**Affiliations:** 1 Obesity and Metabolism Research Unit, United States Department of Agriculture-Agricultural Research Services-Western Human Nutrition Research Center, 430 West Health Sciences Drive, Davis, CA 95616 USA; 2 Department of Nutrition, University of California Davis, Davis, CA USA; 3 Human Studies Unit, United States Department of Agriculture-Agricultural Research Services-Western Human Nutrition Research Center, Davis, CA USA; 4 Immunity and Disease Prevention Research Unit, United States Department of Agriculture-Agricultural Research Services-Western Human Nutrition Research Center, Davis, CA USA; 5 West Coast Metabolomics Center, Genome Center, University of California Davis, Davis, CA USA

**Keywords:** Cardiometabolic disease, dietary guidelines for Americans, Dietary patterns, HEI-2015, Multivariate analysis, Risk prediction

## Abstract

**Background:**

Associations between diet and cardiometabolic disease (CMD) risk may vary in men and women owing to sex differences in eating habits and physiology. The current secondary analysis sought to determine the ability of sex differences in dietary patterns to discriminate groups with or without CMD risk factors (CMDrf) in the adult population and if this was influenced by age.

**Methods:**

Diet patterns and quality were evaluated using 24 h recall-based Healthy Eating Index (HEI-2015) in free-living apparently healthy men (*n* = 184) and women (*n* = 209) 18–65 y of age with BMIs of 18–44 kg/m^2^. Participants were stratified into low- and high-CMDrf groups based on the presence/absence of at least one CMDrf: BMI > 25 kg/m^2^; fasting triglycerides > 150 mg/dL; HDL cholesterol < 50 mg/dL-women or < 40 mg/dL-men; HOMA > 2; HbA1c > 5.7. Sex by age dietary patterns were stratified by multivariate analyses, with metabolic variable associations established by stepwise discriminant analysis.

**Results:**

Diet quality increased with age in both sexes (*P* < 0.01), while women showed higher fruit, vegetable and saturated fat intake as a percentage of total energy (*P* < 0.05). The total-HEI score (i.e. diet quality) was lower in the high-CMDrf group (*P* = 0.01), however, diet quality parameters predicted CMDrf presence more accurately when separated by sex. Lower ‘total vegetable’ intake in the high-CMDrf group in both sexes, while high-CMDrf men also had lower ‘total vegetables’, ‘greens and beans’ intake, and high-CMDrf women had lower ‘total fruits’, ‘whole-fruits’, ‘total vegetables’, ‘seafood and plant-proteins’, ‘fatty acids’, and ‘saturated fats’ intakes (*P* < 0.05). Moreover, ‘dairy’ intake was higher in high-CMDrf women but not in men (sex by ‘dairy’ interaction *P* = 0.01). Sex by age diet pattern models predicted CMDrf with a 93 and 89% sensitivity and 84 and 92% specificity in women and men, respectively.

**Conclusions:**

Sex and age differences in dietary patterns classified participants with and without accepted CMDrfs, supporting an association between specific diet components and CMD risk that differs by sex. Including sex specific dietary patterns into health assessments may provide targeted nutritional guidance to reduce the burden of cardiovascular disease.

**Trial registration:**

ClinicalTrials.gov: NCT02367287. ClinicalTrials.gov: NCT02298725.

**Supplementary Information:**

The online version contains supplementary material available at 10.1186/s40795-021-00432-4.

## Background

Obesity, insulin-resistance, dyslipidemia, and elevated blood pressure represent a cluster of metabolic abnormalities constituting risk factors for cardiometabolic syndrome [1]. The prevalence of the cardiometabolic disease (CMD) increases with age [2], and cardiovascular and metabolic manifestations vary in women and men [3, 4]. As of 2012 25% of the world’s adult population were suffering from this cluster of metabolic dysfunctions [5]. While obesity and diabetes have increased in the Americas, hypertension has had a modest decline between 1980 and 2014, but the rates of these changes reportedly vary by sex [2]. Sex differences in the incidence of heart failure, hypertension, metabolic irregularities in glucose, lipid and cardiac energy metabolism, and endothelial function have all been observed [3, 6, 7]. While not regularly implemented clinically, sex-specific cardiometabolic disease risk factor management presents an opportunity [8]. Among the several factors that influence cardiometabolic disease risk, diet is a modifiable lifestyle parameter that needs to be better understood within a sex-specific context. Case in point, controlled feeding of an isoenergetic Mediterranean diet for 4wks improved plasma lipid profile in both men and women with mild-impairments of cardiometabolic factors, but only men showed significant improvements in glucose stimulated insulin responses [9]. Understanding how habitual diet may influence the development of cardiometabolic disease risk factors (CMDrfs) in a sex by diet by age specific manner may provide clinicians and policy makers guidance to devise sex/age-specific nutritional recommendations, to better manage the prevalence of cardiometabolic disease.

The healthy eating index (HEI) is a tool that measures diet quality as reflected by the recommendations of the Dietary Guidelines for Americans [10]. First developed in 2005, and most recently updated in 2015, the HEI segregates recalled dietary information from either long term food frequency questionnaires or a series of 24 h dietary recalls [11, 12] into subcategories, scored on scales of adequacy or moderation, for putatively healthy and unhealthy food components, respectively. Adequate component scores increase, while moderate component scores decrease with consumption. As a result, the total HEI score represents a multivariate aggregate of diet quality relative to federal dietary guidelines, with scores increasing with diet quality. Elevated HEI scores have been associated with a reduced risk of overall death including cardiovascular disease and cancer [13].

The 2015 HEI adequate dietary components include ‘total fruit’, ‘whole fruit’, ‘total vegetables’, ‘greens and beans’, ‘whole grains’, ‘dairy’, ‘total protein’, ‘seafood & plant proteins’, and ‘fatty acids’, which are recommended to be high in a healthy diet [14]. In contrast, moderate dietary components where consumption is recommended to be limited include ‘refined grains’, ‘sodium’, ‘added sugar’ and ‘saturated fatty acids’ [14]. Hence, a multivariate evaluation of the HEI subscores can be seen as a low-resolution diet pattern analysis that can differentiate diets with similar total HEI scores. A preliminary comparison of diet patterns in women with mild impairment of either glucose homeostasis, lipid metabolism or both [15], to those from a contemporaneous cross-sectional study of men and women [16], indicated that the diet patterns of the metabolically compromised women represented a subset of the general population, supporting an association between diet and the presence of CMD risk factors (CMDrfs). The objective of this secondary analysis was to identify sex differences and sex by age relationships between established CMDrfs and HEI-based diet quality scores and subscores. Associations between subscores and CMDrfs may reveal the effects of nutritional components on CMDrf development and place them in a context easily translatable into individualized nutritional recommendations.

## Methods

### Participants

In the USDA Western Human Nutrition Research Center (WHNRC) Cross-Sectional Nutritional Phenotyping Study (Phenotyping Study; ClinicalTrials.gov: NCT02367287), generally healthy individuals were recruited in Davis, CA starting in May of 2015. Details of study recruitment and participation are contained in a separate report under consideration for publication in *Stress: International Journal on the Biology of Stress* (personal communication, Dr. Kevin Laugero, USDA - WHNRC, Davis CA). Briefly, participants were excluded if they were pregnant or lactating, had recently undergone a minor surgery, recently received antibiotic therapy, had been hospitalized in the past 4 wk., had major surgery in the past 16 wk., were currently taking daily medication for a diagnosed chronic disease, or had known egg allergies (egg white protein was a component of the meal challenge test used to assess insulin sensitivity). Participants were recruited into 18 categories defined by sex, three age (18–33 y, 34–49 y, 50–65 y) and three body mass index (BMI; 18.5–24.99 kg/m^2^, 25–29.99 kg/m^2^, and 30–39.99 kg/m^2^) categories filled at a relatively even rate to balance enrollment across seasons and over 4 years [16]. The final enrolled cohort included men (*n* = 184) and women (*n* = 209) between the ages of 18–66 with BMIs of 18–44 kg/m^2^ (normal to obese).

Anthropometry, body composition, various physiological and psychological test outcomes, and biological specimens (plasma, urine, feces etc.) were collected during two study visits spaced 10–14 days apart. A standardized meal challenge test in all participants allowed the evaluation of postprandial insulin sensitivity [17]. While anthropometry was obtained on all subjects, clinical blood parameters were only determined on a total of 362 individuals. Data were captured in Research Electronic Data Capture (REDCap™) a web-based application hosted by the University California Davis Health System Clinical and Translational Science Center [18]. The study was reviewed and approved by the University of California, Davis, Institutional Review Board. All participants provided written informed consent and received monetary compensation for their participation.

### Dietary assessments

Unannounced 24-h dietary recalls were completed using the Automated Self-Administered 24-h dietary recall system to obtain information on participant habitual diets [12]. Each participant was provided orientation to the assessment tool and completed a training recall with staff assistance. Data from at least two completed dietary assessments were used to calculate Healthy Eating Index (HEI) Scores for each participant according to established guidelines [10, 13] using SAS 9.3 statistical software. The HEI scoring guidelines are outlined in Supplemental Table 1.

### Clinical assessments

Protocols used to obtain anthropometric and metabolic measures were previously described [16]. Fasting values for glucose and insulin were used to calculate the homeostatic model assessment of insulin resistance (HOMA) [19].

Insulin resistance was also estimated from the postprandial response to a mixed macronutrient meal challenge test (MCT). The MCT contained palm oil, sucrose, and pasteurized liquid egg white protein as the main ingredients with a composition of fat (60 cal%): carbohydrate (28 cal%): protein (12 cal%) and 62.5% moisture [16]. Postprandial insulin to glucose relationship cutoffs indicative of insulin resistance were established by comparison to classic determination using an oral glucose tolerance test (OGTT) in the independent cohort of women in the controlled feeding intervention trial (*n* = 44) using the approach of Matsuda and DeFronzo [20]. For this validation, MCTs and OGTTs were performed within 2 days of each other on three separate visits before and at 3 and 8 weeks of the intervention. The prediction of the presence or absence of insulin resistance using independent OGTT or MCT measurements achieved 86% accuracy supporting the equivalency of these measurements. The full validation of this modified Matsuda index calculation is being prepared for publication.

The Framingham lipid-based 10 y cardiovascular disease risk was calculated for a subset of participants based on the American Heart Association and the American College of Cardiology [21]. Available online tool (http://tools.acc.org/ASCVD-Risk-Estimator-Plus/#!/content/terms/). This estimation used sex, age, total cholesterol, HDL-cholesterol, systolic blood pressure, blood pressure lowering medication use, diabetes status, and smoking status from participants ≥30y old (*n* = 120 women; *n* = 107 men).

### CMD-risk factor prevalence group classification

Participants were a priori stratified into two groups based on CMDrf presence (high CMDrf) or absence (low CMDrf). To be classified into the high CMDrf group, participants had to have at least one of the following risk factors [20]: BMI 25–44 kg/m^2^; fasting triglyceride concentrations > 150 mg/dL; HDLc < 50 mg/dL-women or < 40 mg/dL-men; HOMA > 2 or HbA1c ≥5.7 and < 6.5%. Participants without any of these risk factors were classified into the low CMDrf group.

### HEI-based risk factor prevalence prediction

**Fig. 1:**
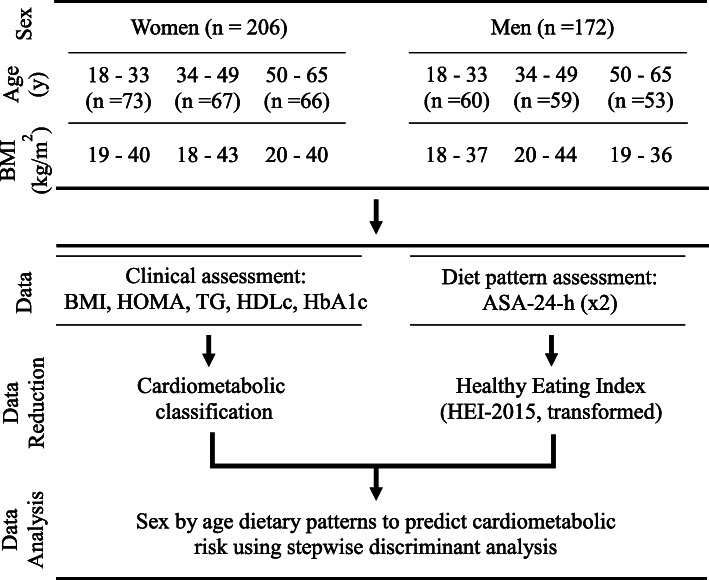
Graphical summary of study design. WHNRC Nutritional Phenotyping study Cohort characteristics along with the data and data analysis workflow covered in the current study. Cohort ethnic demographics match that of the 2010 population census of California

An overview of the study design is presented in Fig. 1. To assess the ability of HEI-dietary components to predict CMDrf group association, both Fisher’s linear discriminant analyses and logistic regression were evaluated [22]. A stepwise discriminant analysis was ultimately used to stratify participants into low and high CMDrf groups based on a minimum set of HEI-components. The analysis of covariance test for the group variable (F ratio and prob.>F statistic) was an indicator of its discriminatory significance. Both linear discriminant analysis (LDA) and a quadratic discriminant analysis (QDA) were investigated respectively.

$$ {D_i^{\ast}}^2\left(\boldsymbol{x}\right)={\left(\boldsymbol{x}-{\overline{\boldsymbol{x}}}_{\boldsymbol{i}}\right)}^{\prime }{\boldsymbol{S}}_{\boldsymbol{p}}^{-\mathbf{1}}\left(\boldsymbol{x}-{\overline{\boldsymbol{x}}}_{\boldsymbol{i}}\right)\ \left(\mathrm{LDA}\right) $$

$$ {D_i^{\ast}}^2\left(\boldsymbol{x}\right)={\left(\boldsymbol{x}-{\overline{\boldsymbol{x}}}_{\boldsymbol{i}}\right)}^{\prime }{\boldsymbol{S}}_{\boldsymbol{i}}^{-\mathbf{1}}\left(\boldsymbol{x}-{\overline{\boldsymbol{x}}}_{\boldsymbol{i}}\right)+\boldsymbol{\ln}\left|{\boldsymbol{S}}_{\boldsymbol{i}}\right|\ \left(\mathrm{QDA}\right) $$

where *Di* is the score on the discriminant function *i*, *x* is the standardized values of the discriminant variables, *Sp* is the estimated common covariance matrix and *Si* is the estimated covariance matrix for group *i*. Each observation ***x*** is estimated to each *i* group′s multivariate mean (centroid) using Mahalanobis distance $\left(\boldsymbol{x}-{\overline{\boldsymbol{x}}}_{\boldsymbol{i}}\right)$, which considers the correlation structure of the data and the individual scales. Classification then depends on either a linear or quadratic combination of the discriminating variables for each group to produce a probability membership, with assignment based on the highest probability.

For sex by age model building, data were divided into a 75/25 training:validation set splits to avoid over-fitting and minimize model reliance on sample selection. Validation sets were selected to balance training set representation and used to test model fit probabilities (Entropy *R*^*2*^). Discriminant equation expected error rates (%) were estimated as the discriminant function classification accuracy over the maximum accuracy obtained from training sample data. A randomized permutation test was used to simulate the accuracy score null distribution to determine if the classification was above-chance [23]. On each iteration of the permutation test, the no-risk and risk labels were randomly reassigned within each participant, and the cross-validated accuracy was recalculated. This was repeated 2500 times to create a distribution of classifier accuracy scores expected under the assumption that risk classifications blocks are exchangeable (Supplemental Fig. 1).

Models were evaluated using confusion matrices, eigenvalues, canonical correlations, likelihood ratios and *p*-values. Model sensitivity (the ability to predict the condition when the condition is present) and specificity (the ability to predict the absence of the condition when the condition is not present) were calculated for each model from confusion matrices. The discriminant power was estimated from the area under the receiver operator characteristic curve (AUC_ROC_). Models with excellent (AUC_ROC_ ≥ 0.9) or good (0.8 > AUC_ROC_ > 0.9) performance were considered. Sex and age categories were initially evaluated as covariates, either alone or in combination, in the CMDrf group models with all HEI-components and neither was significant (Supplemental Table 5). Stepwise discriminant models were generated using all HEI-components for each sex (women, *n* = 206; men, *n* = 172) at three different age categories (18 to 33 y, *n* = 133; 34 to 49 y, *n* = 126; and 50 to 65 y., *n* = 119. To enhance confidence in the HEI-based CMDrf group stratification approach, we also applied the constructed models to an available data set from an independent cohort of women (*n* = 44) enrolled for the presence of CMDrfs that had participated in a controlled feeding intervention trial [15].

### Statistical analysis

Data normality was assessed using the Shapiro-Wilk test and Q-Q plots, and transformations applied as necessary prior to effect testing or modeling efforts. Total-HEI scores were normally distributed. The 13 HEI-components were transformed using a marginal rank-based inverse normal transformation scaled from 0 to 1 [24], but remained non-normal. Mean differences between predicted CMDrf group BMI, lipid and glucose profiles and Total HEI-components were examined using Student’s t-test or chi-square test (for categorical variables) with comparison across age categories and sexes. Age by sex interactions were identified using either a Tukey-Kramer or Wilcoxon signed-rank multicomparison tests for parametric and non-parametric data, respectively. Pearson correlations were used to identify associations among HEI components. Correlation strength was considered strong (|r| ≥ 0.7), moderate (0.7 > |r| ≥ 0.4) or weak (0.4 > |r| ≥ 0.1) [15]. All analyses were performed using JMP Pro version 14.0 (SAS Institute, Cary, North Carolina).

## Results

### Relationship between HEI-components

Correlations between the HEI component and total scores were evaluated to determine the amount of unique information contained in each HEI-components. The total HEI score had moderate-to low correlations with the individual HEI-component scores, but the HEI-components showed many significant correlations (*P* < 0.05, *r >* 0.1; *n* = 378; Supplemental Table 3). Of those, strong correlations were seen between ‘total fruits’ and ‘whole fruits’ (*r =* 0.87) and ‘fatty acids’ and ‘saturated fats’ (*r =* 0.73). Moderate correlations were seen between ‘greens and beans’ and both ‘total vegetables’ (*r =* 0.63) and ‘seafood and plant proteins’ (*r =* 0.45), ‘total vegetables’ and ‘added sugars’ (*r =* 0.43), and between ‘dairy’ and ‘fatty acids’ (*r =* − 0.42), and between ‘total protein’ and ‘seafood and plant proteins’ (*r =* 0.40). All other observed correlations were weak (*r <* 0.4).

### Dietary differences with age, sex and cardiometabolic risk factors

Overall, the total HEI scores were similar between sexes, and were higher (*P* < 0.01) in those 50 to 65 y compared to those 18 to 49 y, regardless of CMDrf group association. However, other sex and age associations with CMDrfs differed. A higher total-HEI score was associated with lower HOMA in women but lower BMI in men. In addition, a higher total-HEI score was associated with lower BMI and HOMA in the young and middle-aged groups, but only a lower BMI in older individuals.

Numerous sex-dependent differences in HEI-components were detected. Women had higher scores for ‘total vegetables’ and ‘whole fruit’ (*P* < 0.05) and a lower ‘saturated fat’ score (*P* = 0.03) than men (Supplemental Table 2). In women, higher scores also approached significance for ‘total fruits’ (*P* = 0.07), ‘dairy’ (*P* = 0.09), and ‘refined grain’ (*P* = 0.1), as did lower scores for ‘total protein’ (*P* = 0.06) and ‘added sugars’ (*P* = 0.06). Men aged 18 to 33 y had lower ‘total fruit’ and ‘whole fruit’ scores than other age categories (*P* < 0.01), while women 50 to 65 y had higher ‘refined grain’ scores and men 50 to 65 y had higher ‘saturated fats’ scores than other age groups (*P* < 0.05). Women and men 50 to 65 had higher ‘whole grain’ and ‘sodium’ scores than other age groups (*P* < 0.01). Scores for ‘greens and beans’, ‘seafood and plant proteins’, and ‘fatty acids’ showed no differences between sex or age.

### CMDrf group classification

Of the 393 participants, 286 were stratified into the high CMDrf group with either 1 (*n* = 71; *n* = 62), 2 (*n* = 39; *n* = 31), or 3+ (*n* = 47; *n* = 36) risk factors for women and men, respectively. Notably participant selection was stratified to provide balanced sex and age coverage of normal, overweight and obese participants, thereby may over sample the high CMDrf group from the geographic area. Regardless, only 25% (*n* = 99) of participants had BMI > 25 kg/m^2^ as a unique risk factor. Of the 31 participants without clinical blood measures, 25 were classified in the high CMDrf group by BMI. Of the participants with BMI and clinical blood measurements, only 5% were classified as high risk with BMI < 25 kg/m^2^. Therefore, of the 6 participants classified in low CMDrf group based on BMI without supporting clinical measurements, it is estimated that 2 could be misclassified. This would represent a misclassification rate of 0.5% and was deemed acceptable. With these caveats, the phenotyping cohort was stratified into low- (*n* = 107; ~ 27%) and high (*n* = 286; ~ 73%) CMDrf groups that differed (*P* < 0.01) in BMI, HDLc, triglycerides (TG) and HOMA (Table 1, Supplemental Table 4). Sex differences in the prevalence of overweight and obese conditions were not significant. In the low CMDrf group, participants aged between 50 to 65 y had higher HDLc (*P* < 0.01) than 18 to 49 y olds (72 vs 63 mg/dL). TG was not higher (*P* = 0.10) in the high CMDrf group in participants aged between 34 to 65 y compared to 18 to 33 y olds (115 vs 90 mg/dL). Only 15 participants were tobacco-users and were not associated with specific CMDrf groups.

**Table 1:** Comparison of clinical parameters used to stratify cardiometabolic risk groups^a^

Risk factors	Cardiometabolic risk				*P-value*	
	Low-risk (*n =* 107)		High-risk (*n =* 286)			
	Mean ± SE	Criteria (%)	Mean ± SE	Criteria (%)	Risk by age	Risk
BMI (kg/m^2^)	22.5 ± 0.42	0	29.3 ± 0.24	87%	0.56	< 0.01
HDLc _women_ (mg/dL)	74.0 ± 1.91	0	55.5 ± 1.14	42%	< 0.01	< 0.01
HDLc _men_ (mg/dL)	57.7 ± 1.89	0	46.0 ± 1.23	45%	0.04	< 0.01
TG_fasting_ (mg/dL)	73.4 ± 4.25	0	107 ± 2.93	14%	0.12	< 0.01
HOMA	1.08 ± 0.23	0	2.63 ± 0.14	45%	0.82	< 0.01
HbA1c (%)	5.24 ± 0.04	0	5.32 ± 0.02	9%	0.81	0.11

^a^Study participants were classified a priori for a cardiometabolic outcome. High risk was based on at least one of the following criteria: BMI (kg/m^2^) of 25–44; fasting triglycerides > 150 mg/dL; HDLc < 50 mg/dL-women or < 40 mg/dL-men; HOMA > 2; HbA1c ≥5.7 and < 6.5. Low risk was based on the absence of all risk factors. The interactions of the risk by sex or 3 components (age, sex and risk) was not significant. Values are mean and standard errors (SE)

### Dietary score differences between CMDrf groups

Of the 393 participants, HEI scores were calculated for 378 who completed two or three 24-h recalls. The high CMDrf group had lower total HEI-score than the low CMDrf group (total HEI-score 60 vs. 66, respectively; *P* < 0.01). Similarly, the high CMDrf group had lower scores for ‘total fruits’, ‘whole fruits’, ‘total vegetables’, ‘greens and beans’, ‘seafood and plant proteins’, ‘fatty acids’, and ‘saturated fats’.

**Fig. 2:**
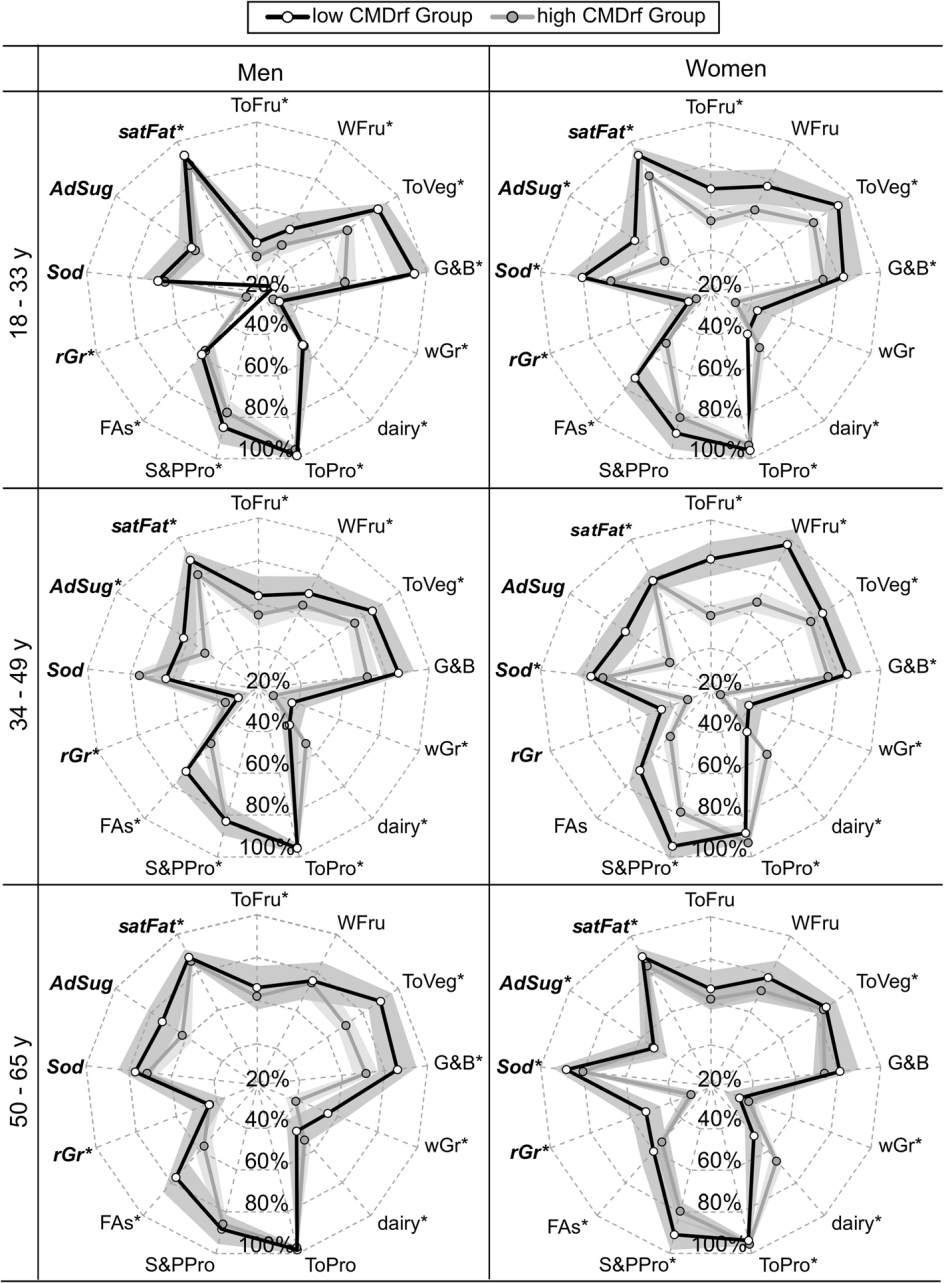
Diet patterns by sex and age for low and high cardiometabolic risk factors (CMDrf). Radar graph depicting dietary patterns of quality according to Healthy Eating Index-2015 (HEI) for a low (*n =* 106) and high (*n =* 272) CMDrf in women and men by age in a cross-sectional study. HEI-component scores are expressed as a percentage of their maximum score, with scores increasing with diet quality. Each point represents the mean ± standard error of means. Diet components in bold-italic are recommended to be eaten in moderation. * The symbol represents the HEI-components included in the predicted CMDrf models. Abbreviation of HEI-components are total-vegetables (ToVeg); saturated fat (satFat); total protein (ToPro); refined-grains (rGr); ‘fatty acids’ (FAs); ‘greens and beans’ (G&B); whole-grain (wGr) total-fruit (ToFru); sea-food and plants (S&PPro); whole-fruits (WFru); ‘added sugars’ (AdSug; *n =* 3); ‘sodium’ (Sod)

Sex-specific differences between high and low CMDrf groups in HEI-components of ‘total fruits’, ‘whole fruits’, ‘total vegetables’, ‘greens and beans’, ‘dairy’, ‘seafood and plant proteins’, ‘sodium’, ‘fatty acids’, and ‘saturated fats’ scores were observed (*P* < 0.05; Fig. 2). In women, the high CMDrf group had lower ‘total fruits’, ‘whole fruits’, ‘seafood and plant proteins’, ‘total-vegetables’, ‘sodium’, ‘fatty acids’, and ‘saturated fats’ and higher ‘dairy’ scores. In contrast, men in the high CMDrf group only showed lower ‘total vegetables’ and ‘greens and beans’ scores. ‘Whole grain’, ‘refined grain’, and ‘added sugars’ did not differ between CMDrf groups.

### HEI-component-based prediction of CMD-risk factor presence

As multiple sex by age differences in HEI-component scores were identified (Supplemental Table 2), models were built to assess age x sex categories (Supplemental Tables 5, 6, and 7). HEI-component-based CMDrf group prediction was excellent for women, and good for men across age groups. (Table 2, Supplemental Fig. 2). The frequency of HEI-component inclusion by the stepwise discriminant analysis in the six age x sex models were ‘dairy’ = ‘total vegetables’ = ‘saturated fats’ (*n* = 6; 100%) > ‘greens and beans’ = ‘total proteins’ = ‘refined grain’s = ‘fatty acids’ (*n =*5; 83%) > ‘whole grain’s = ‘total fruits’ = ‘seafood and plant proteins’ (*n* = 4; 66%) > ‘whole fruits’ = ‘added sugars’ = ‘sodium’ (*n* = 3; 50%) (Supplemental Table 8).

**Table 2:** HEI-2015 component stepwise discriminant models for cardiometabolic risk groups in women and men by age category

Age (y)	Model components	Percent Predicted (%)		AUC	Entropy ***R***^**2**^	Prob > F
		Low-risk (***n*** = 106)	High-risk (***n*** = 272)			
***Women***						
18 to 33	Dairy, ToVeg, satFat, ToFru, ToPro, rGr, FAs, G&B, AdSug, Sod	93	90	0.93	0.66	0.10
34 to 49	Dairy, ToVeg, satFat, ToFru, ToPro, G&B, wGr, S&PPro, WFru, Sod	100	98	0.96	0.75	0.03
50 to 65	Dairy, ToVeg, satFat, ToPro, rGr, FAs, wGr, S&PPro, AdSug, Sod	100	100	0.92	0.85	0.02
***Men***						
18 to 33	Dairy, ToVeg, satFat, ToPro, rGr, FAs, G&B, wGr, ToFru, S&PPro, wFru	92	92	0.89	0.86	0.04
34 to 49	Dairy, ToVeg, satFat, ToPro, rGr, FAs, wGr, ToFru, S&PPro, wFru, AdSug	100	95	0.83	0.85	0.07
50 to 65	Dairy, ToVeg, satFat, rGr, FAs, G&B, wGr, ToFru	100	96	0.89	0.95	0.05

HEI-components ranked by frequency of appearance in models: dairy (*n =* 6); total-vegetables (ToVeg; *n =* 6); saturated fat (satFat; *n =* 6); total protein (ToPro; *n =* 5); refined-grains (rGr; *n =* 5); ‘fatty acids’ (FAs; *n =* 5); ‘greens and beans’ (G&B; *n =* 5); whole-grain (wGr; *n =* 4); total-fruit (ToFru; *n =* 4); sea-food and plants (S&PPro; *n =* 4); whole-fruits (WFru; *n =* 3); ‘added sugars’ (AdSug; *n =* 3); ‘sodium’ (Sod; *n =* 3). AUC = area under the curve

The dietary component CMDrf group classification accuracy showed high sensitivity (91% accuracy) within sex and age (Table 3). However, model specificity was age dependent, increasing with age: younger-age (78%) < middle-age (85%) < older-age (> 90%). The predicted vs a priori CMDrf misclassification rate was only 10% overall, ranging from 8 to 14% across sex and age groups, with misclassification highest in the youngest groups (Supplemental Table 9). Similarly, these diet-based models for women classified the independent group of overweight to obese women from the controlled feeding intervention with 100, 87 and 100% sensitivity for young, mid and older age groups, respectively. Except for lower ‘fatty acids’ scores in the controlled feeding intervention (*P* = 0.04), HEI-components were similar in high CMD-risk groups across the two studies (Supplemental Table 10).

**Table 3:** Accuracy of cardiometabolic risk prediction by HEI-2015 components

Stepwise discriminate model	Classification Accuracy (%)	
	Phenotyping	
	Low CMDrf	High CMDrf
***Women***		
18 to 33 y	71	91
34 to 49 y	80	96
50 to 65 y	100	93
***Men***		
18 to 33 y	85	93
34 to 49 y	92	85
50 to 65 y	100	89

‘Dairy’, ‘total vegetables’ and ‘saturated fats’ were the HEI-components common to all diet-based CMDrf prediction models across sex and age. The average HEI-component profiles of the predicted CMD-risk groups are shown in Supplemental Table 11 and are parallel to the findings reported in Fig. 2.

### Risk factor patterns in diet-predicted high vs low CMDrf groups

Finally, we evaluated how HEI-component based CMDrf group prediction segregated the CMD risk factors used for stratification and other clinical parameters associated with cardiometabolic health. As expected, BMI, fasting insulin, and HOMA were higher in both sexes across all age categories in the high CMDrf group (*P* < 0.05; Table 4). Postprandial insulin resistance was also higher in high CMDrf groups in both sexes (*P* < 0.01), but was higher in men than women (*P* < 0.01). The lipid profile of the predicted high CMDrf group was characterized by a lower HDLc and higher total cholesterol, LDL cholesterol (LDLc), and fasting TG in both sexes at all ages (*P* < 0.05). The HDLc concentration increased with age in the low CMDrf group in women (68, 72, 82 mg/dL respectively for age category), but not in men, while remaining low in the high CMDrf group across age and sex (*P* = 0.01; Table 4). For participants over 30y of age, the average Framingham risk (%) was also higher (*P* = 0.03) in 5.5% in the high compared to 4% in the low CMDrf groups, but sex by risk interactions were not significant (*P* = 0.07).

**Table 4:** Metabolic profile in women and men (*n* = 393) by predicted stepwise discriminative cardiometabolic risk groups^a^

Metabolic variables	sex	Phenotyping study		SE	*P-value*	FDR^d^
		Low CMDrf	High CMDrf		Risk	
BMI (kg/m2)	women	23.6	29.1	0.44	**< 0.01**	**< 0.01**
	men	23.0	28.4	0.58		
LDLc (mg/dL)	women	99.5	110	3.51	**< 0.01**	**< 0.01**
	men	93.9	114	3.62		
HDLc (mg/dL)	women	70.7	56.9	1.44	**< 0.01**	**< 0.01**
	men	56.8	47.2	1.98		
Cholesterol (mg/dL)	women	177	183	3.53	**< 0.01**	**0.02**
	men	161	179	3.72		
Triglycerides (mg/dL)	women	70.0	102	6.18	**< 0.01**	**< 0.01**
	men	81.2	108	6.83		
NEFA (mmol/L)	women	0.32	0.36	0.01	0.06	0.08
	men	0.32	0.33	0.01		
Insulin (pmol/L)	women	110	155	9.58	**0.03**	**0.05**
	men	104	133	9.36		
Glucose (mg/dL)	women	91.3	95.4	1.17	0.24	0.28
	men	94.9	96.9	1.36		
HOMA	women	1.32	2.41	0.24	**< 0.01**	**0.01**
	men	1.50	2.54	0.22		
MCT Matsuda Index^b^	women	14.6	9.44	1.22	**< 0.01**	**< 0.01**
	men	17.0	12.6	1.96		
HbA1C %	women	5.26	5.31	0.05	0.28	0.69
	men	5.27	5.31	0.04		
Systolic (mm Hg)	women	117	118	1.11	**0.05**	0.10
	men	118	122	1.25		
Diastolic (mm Hg)	women	65	68	1.01	**0.04**	**0.05**
	men	66	72	1.32		
Framingham risk (Log)^c^	women	0.79	1.05	0.09	**0.03**	**0.05**
	men	1.94	1.70	0.15		

^a^Mean differences between predicted cardiometabolic risk group BMI, lipid and glucose profiles were examined using Student’s t-test. The model interactions age, sex with predicted risk was not significant

^b^Meal challenge test Matsuda Index cut-off of < 8.8 is indicative of insulin resistance

^c^The Risk Calculator estimate 10-year and lifetime risks for atherosclerotic cardiovascular disease (ASCVD), provided by the American Heart Association and the American College of Cardiology. Non-normally distributed risk (%) was log-transformed

^d^False discovery rate *post-hoc p*-adjustment

## Discussion

The prevalence and manifestation of CMD and its risk factors are known to differ between the sexes [3] and differentially change with age [25] depending on lifestyle habits [26]. Diet constitutes an important modifiable variable associated with CMD risk [26] that also varies by sex [27] and age [28]. How diet patterns are associated with the presence of CMD risk factors, and how such associations differ between the sexes are poorly understood. Current dietary recommendations follow nutritional goals that focus on meeting nutrient needs, while limiting the intake of ‘added sugars’, ‘saturated fats’, and ‘sodium’. These recommendations do not capture potentially excessive intakes of the adequacy components (e.g. dairy) and are not stratified by sex [14]. If sex-specific interactions between habitual diet and physiology influence cardiometabolic health, modification of dietary recommendations may be warranted.

Dietary habits can influence health. Indeed, we found that a higher diet quality was associated with beneficial anthropometric and metabolic characteristics. Similarly, previous studies have indicated that diet quality assessed using diet quality indexes is negatively associated with cardiometabolic risk factors [29–32]. For instance, a study of 4097 US adults over 20 y of age identified inverse associations between the HEI-2010 total-score and BMI, triglycerides, TG/HDLc ratio and the presence of comorbidities [33]. Unreported in these previous works, we also observed that HEI component mixture complexities did not co-vary with the total diet quality, but shifted with sex and age. Considering that diet patterns change with age, as do energy intakes and requirements [34], these findings are perhaps not surprising. However, the presence/absence of CMD risk factors in adult men and women could be successfully determined using HEI-2015 components alone, but required both separation of the sexes, and stratification by age greatly enhanced the models. ‘Dairy’, ‘total vegetables’, and ‘saturated fats’ were the only diet components common in all sex/age CMDrf presence/absence discrimination models. In women, the importance of components in order of model appearance was ‘dairy’, ‘total-proteins’, ‘saturated fats’, ‘sodium’, ‘total vegetables’. However, in men the predictive dietary factors were ranked in the order of ‘dairy’, ‘fatty acids’, ‘saturated fats’, ‘greens and beans’, ‘total vegetables’, ‘refined grains’. Notably, the components having the lowest correlations with the total score, i.e. ‘dairy’, had the highest predictive value for CMD risk factor presence/absence that manifested in a sex and age specific manner.

Dairy comprises a wide range of food products with distinctive macronutrients with varying and reportedly contradictory impacts on cardiometabolic health [35]. In our results, ‘dairy’ intake was the only adequacy HEI-component positively correlated to the presence of CMD risk factors in all women, it was not in men. Two recent publications describe relationships with dairy intake that support these results. First, a prospective study in U. S urban adults (30–64 y; *n* = 1371) reported an inverse association between ‘dairy’ fat intake to obesity in men, but a positive relationship with dyslipidemia in women [36]. Second, in a longitudinal study of French-adults (28-60y; *n* = 588), higher consumption of ‘dairy’ products was positively associated with HDL-C and inversely associated with fasting glucose in men, but in women, higher ‘dairy’ consumption was positively related to BMI, waist circumference and TGs [37].

Despite the importance of ‘dairy’ in these models, it is critical to highlight that dairy intake alone does not predict the presence/absence of CMD risk factors. Diet pattern analysis plays a unique role in assessing the relationship between diet and disease because it is more strongly related to the risk of disease than individual foods [38]. Indeed ‘dairy’ was inversely associated with ‘fatty acids’ and ‘saturated fatty acids’ components. While the reduction of saturated fatty acids (SFAs) in the diet is recommended to prevent CMD risk factors [39], the major source of dietary SFAs is provided by the low-nutritive value of highly processed foods, followed by animal food-based products [40]. Therefore, complex dietary factors interplay to make of dietary pattern that may influence many chronic diseases.

It is also important to consider that the development of some CMD risk factors (e.g. insulin resistance and atherosclerosis) may take time [41], and the lag between insult and injury may underlay the lower predictive performance we observed in younger individuals. Similarly, dietary habits may become more stable with time, allowing the identification of stronger associations between habitual diet and CMD risk factors in older populations. Regardless, consideration of sex and age may advance the goal of developing public health targets by promoting healthful eating habits.

### Limitations

The cross-sectional study design results in associations that cannot support causal inferences between HEI-2015 and cardiometabolic status. In addition, the restricted geographical residence, income, and educational level of the participants may reflect dietary habits. Furthermore, ethnicity was primarily represented by~ 70% white participants. The HEI-2015 calculation used two or three 24-h dietary recalls which are prone to error due to their reliance on the participants’ ability to recall and accurately self-report dietary intake, which may lead to under- or over-reporting. However, to minimize error in these measurements a training recall was performed by all participants with study staff before the at-home recalls used in this report. In addition, these short term assessments may not fully reflect the dietary habits of an individual over time. However, the use of the HEI to collapse the data into a diet pattern likely attenuates these errors, and diet patterns are reportedly stable for up to 5 yrs. [42]. In addition, as the presentation of metabolic risk associated with dietary habits is likely associated with the duration of those practices, the ability of diet to predict the presence of CVD-risk factors would be expected to change with age. Finally, 24 h diet recalls may not be stable predictors of longer term dietary patterns.

## Conclusions

In the current study, using diet patterns described by the HEI-2015 total and subscores we found that: 1) a total HEI-score > 64 (of 100 perfect quality diet score) was associated with the absence of CMD risk factors; 2) HEI component scores yielded better CMDrf predictions than the total scores; 3) stratification of HEI component based CMDrf predictions performed best stratified by sex and age. These results suggest that integrating HEI-2015 subscores along with total HEI-scores into clinical assessments may provide preventative strategies for CMD risk factor development, and sex/age- specific diet-based intervention strategies to enhance the prevalence of a healthy cardiometabolic profile/phenotype.

## Supplementary Information

**Additional file 1: Supplemental Fig. 1.** Permutation test. Simulated distribution of *p*-values (A) and entropy R-square (B) for cardio-metabolic risk (*n* = 2500) under the null distribution of discriminatory HEI-components.

**Additional file 2: Supplemental Fig. 2.** ROC analysis**.** Receiver operational curves of the HEI-2015 component**.** HEI were selected by stepwise discriminant models for cardiometabolic risk groups.

**Additional file 3: Supplemental Table 1.** HEI-2015 Components and Scoring Standards. Description of the HEI scoring system.

**Additional file 4: Supplemental Table 2.** HEI-2015 in a cross-sectional study**.** Mean values of HEI-2015 components in women and men of three different ages categories in the WHNRC Nutritional Phenotyping Study.

**Additional file 5: Supplemental Table 3**. Correlations of HEI-components. Pearson correlation of the HEI-2015 components of the WHNRC Nutritional Phenotyping Study cohort.

**Additional file 6: Supplemental Table 4.** Prevalence of metabolic variables in a cross-section study. Number of participants with high risk metabolic variable and their proportion (%) in the WHNRC Nutritional Phenotyping study high and low CVD-risk groups.

**Additional file 7: Supplemental Table 5.** Discriminant model of cardiometabolic risk. Performance of discriminant models of cardiometabolic risk groups using all HEI-2015 components.

**Additional file 8: Supplemental Table 6.** Nominal logistic regression of cardiometabolic risk. Performance of nominal logistic regression of cardiometabolic risk groups using all HEI-2015 components.

**Additional file 9: Supplemental Table 7.** Nominal logistic regression of cardiometabolic risk using selected HEI-2015 components. Performance of nominal logistic regression of cardiometabolic risk groups using selected HEI-2015 components.

**Additional file 10: Supplemental Table 8.** Stepwise discriminant analysis. HEI-2015 component selection in the WHNRC Nutritional Phenotyping study cohort by stepwise discriminant analysis.

**Additional file 11: Supplemental Table 9**. Comparison of actual vs predicted cardiometabolic risk in a phenotyping study. Stepwise discriminant analysis with the total population or a modeling using women and men by age category.

**Additional file 12: Supplemental Table 10.** Evaluation of the predictive model in an independent study of overweight women. Predicted healthy eating index (HEI)-components of high- cardiometabolic risk comparing a cross-sectional and targeted overweight study in women.

**Additional file 13: Supplemental Table 11.** Comparison of actual vs predicted HEI-components. HEI-components by predicted stepwise discriminative cardiometabolic risk in a cross-sectional study.

## Data Availability

The datasets used and/or analyzed during the current study is a component of a larger study that will be posted together at a later date. In the meantime, the data is available from the corresponding author on reasonable request.

## Abbreviations

CMD: Cardiometabolic disease
CVD: Cardiovascular disease
DGA: Dietary guidelines for Americans
HbA1c: Glycated hemoglobin
HEI: Healthy eating index
HDLc: High-density lipoprotein cholesterol
HOMA: Homeostatic model of assessment - insulin resistance
iMAPS: Individual Metabolism and Physiological Signatures
MCT: Meal challenge test
TC: Total cholesterol
TG: Triglycerides
WHNRC: Western Human Nutrition Research Center
