# Supplementary material for: Healthy eating index patterns in adults by sex and age predict cardiometabolic risk factors in a cross-sectional study
Source: BMC Nutr. 2021 Jun 22;7:30. doi: 10.1186/s40795-021-00432-4 (PMC8218401; doi:10.1186/s40795-021-00432-4)
Supplement: Supplementary file 1 — Additional file 1: Supplemental Fig. 1. Permutation test. Simulated distribution of p-values (A) and entropy R-square (B) for cardio-metabolic risk (n = 2500) under the null distribution of discriminatory HEI-components. [file 40795_2021_432_MOESM1_ESM.docx]

| A)  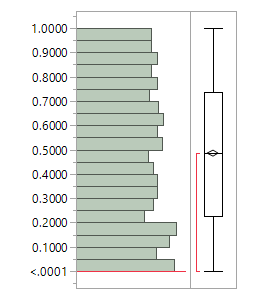  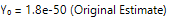   \| **Test** \| **p-Value** \| \| --- \| --- \| \| Y ≥ \|Y₀\| \| 1.0000 \| \| Y ≤ Y₀ \| <.0001* \| \| Y ≥ Y₀ \| 1.0000 \| | B)  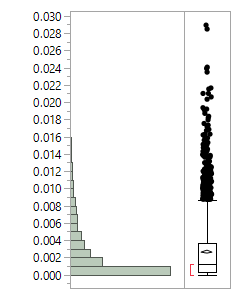  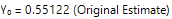   \| **Test** \| **p-Value** \| \| --- \| --- \| \| Y ≥ \|Y₀\| \| <.0001* \| \| Y ≤ Y₀ \| 1.0000 \| \| Y ≥ Y₀ \| <.0001* \| |
| --- | --- | --- | --- | --- | --- | --- | --- | --- | --- | --- | --- | --- | --- | --- | --- | --- | --- |

**Supplemental Figure 1**. Simulated Distribution of p-values (A) and Entropy R-square (B) for cardio-metabolic risk (n=2,500) under the Null Distribution of discriminatory HEI-components.

The observed p-value and R-square were significantly different from the simulated null distribution demonstrating that CMD-risk prediction using HEI-components is not random.
