# Supplementary material for: Healthy eating index patterns in adults by sex and age predict cardiometabolic risk factors in a cross-sectional study
Source: BMC Nutr. 2021 Jun 22;7:30. doi: 10.1186/s40795-021-00432-4 (PMC8218401; doi:10.1186/s40795-021-00432-4)
Supplement: Supplementary file 2 — Additional file 2: Supplemental Fig. 2. ROC analysis. Receiver operational curves of the HEI-2015 component. HEI were selected by stepwise discriminant models for cardiometabolic risk groups. [file 40795_2021_432_MOESM2_ESM.docx]

| **Women 18-33 y** | **Men 18-33 y** |
| --- | --- |
| 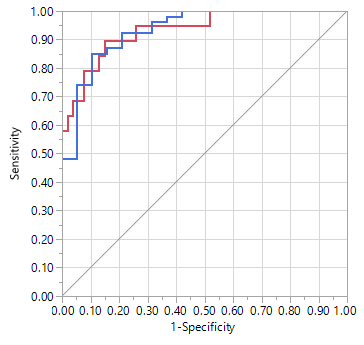   \|  \| **risk factor** \| **Area** \| \| --- \| --- \| --- \| \|  \| Low-risk \| 0.9337 \| \|  \| High-rik \| 0.9337 \| | 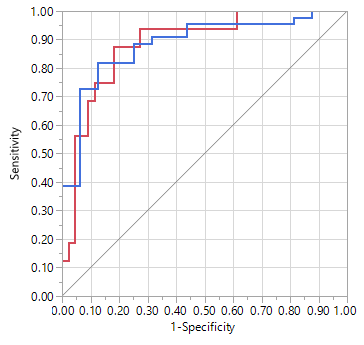   \|  \| **risk factor** \| **Area** \| \| --- \| --- \| --- \| \|  \| Low-risk \| 0.8849 \| \|  \| High-risk \| 0.8849 \| |
| **Women 34-49 y** | **Men 34-49 y** |
| 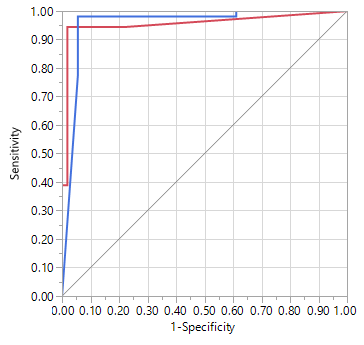   \|  \| **risk factor** \| **Area** \| \| --- \| --- \| --- \| \|  \| Low-risk \| 0.9558 \| \|  \| High-risk \| 0.9558 \| | 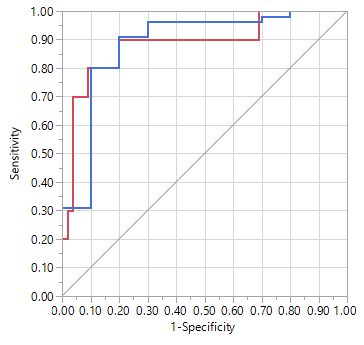   \|  \| **risk factor** \| **Area** \| \| --- \| --- \| --- \| \|  \| Low-risk \| 0.8263 \| \|  \| High-risk \| 0.8263 \| |
| **Women 50-65 y** | **Men 50-65 y** |
| 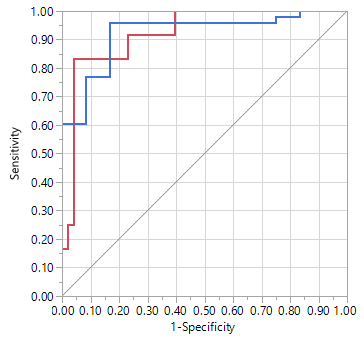   \|  \| **risk factor** \| **Area** \| \| --- \| --- \| --- \| \|  \| Low-risk \| 0.9219 \| \|  \| High-risk \| 0.9219 \| | 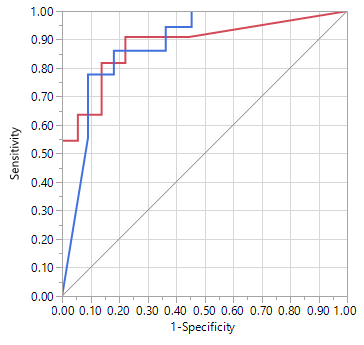   \|  \| **risk factor** \| **Area** \| \| --- \| --- \| --- \| \|  \| Low-risk \| 0.8819 \| \|  \| High-risk \| 0.8819 \| |
|  |  |

**Supplemental Figure 2**. Receiver operational curves of the HEI-2015 component selected by stepwise discriminant models for cardiometabolic risk groups
