# Supplementary material for: Healthy eating index patterns in adults by sex and age predict cardiometabolic risk factors in a cross-sectional study
Source: BMC Nutr. 2021 Jun 22;7:30. doi: 10.1186/s40795-021-00432-4 (PMC8218401; doi:10.1186/s40795-021-00432-4)
Supplement: Supplementary file 3 — Additional file 3: Supplemental Table 1. HEI-2015 Components and Scoring Standards. Description of the HEI scoring system. [file 40795_2021_432_MOESM3_ESM.docx]

| **Supplemental Table 1.** HEI-2015^1^ Components and Scoring Standards | | | |
| --- | --- | --- | --- |
| **Component** | **Max. points** | **Standard for maximum score** | **Standard for minimum score of zero** |
| **Adequacy:** | | | |
| Total Fruits^2^ | 5 | ≥0.8 cup eq/1,000 kcal | No Fruit |
| Whole Fruits^3^ | 5 | ≥0.4 cup eq/1,000 kcal | No Whole Fruit |
| Total Vegetables^4^ | 5 | ≥1.1 cup eq/1,000 kcal | No Vegetables |
| Greens and Beans^4^ | 5 | ≥0.2 cup eq/1,000 kcal | No Dark-Green Vegetables or Legumes |
| Whole Grains | 10 | ≥1.5 oz. eq/1,000 kcal | No Whole Grains |
| Dairy^5^ | 10 | ≥1.3 cup eq/1,000 kcal | No Dairy |
| Total Protein Foods^4^ | 5 | ≥2.5 oz. eq/1,000 kcal | No Protein Foods |
| Seafood and Plant Proteins^4,6^ | 5 | ≥0.8 oz. eq/1,000 kcal | No Seafood or Plant Proteins |
| Fatty Acids^7^ | 10 | (PUFAs + MUFAs)/SFAs ≥2.5 | (PUFAs + MUFAs)/SFAs ≤1.2 |
| **Moderation:** | | | |
| Refined Grains | 10 | ≤1.8 ounce eq/1,000 kcal | ≥4.3 ounce eq/1,000 kcal |
| Sodium | 10 | ≤1.1 g/1,000 kcal | ≥2.0 g/ 1,000 kcal |
| Added Sugars | 10 | ≤6.5% of energy | ≥26% of energy |
| Saturated Fats | 10 | ≤8% of energy | ≥16% of energy |
| ^1^ Intakes between the minimum and maximum standards are scored proportionately.  ^2^ Includes 100% fruit juice.  ^3^ Includes all forms except juice.  ^4^ Includes legumes (beans and peas).  ^5^ Includes all milk products, such as fluid milk, yogurt, and cheese, and fortified soy beverages.  ^6^ Includes seafood, nuts, seeds, soy products (other than beverages), and legumes (beans and peas).  ^7^ Ratio of poly- and mono-unsaturated fatty acids (PUFAs and MUFAs) to saturated fatty acids (SFAs). | | | |
