# Supplementary material for: Healthy eating index patterns in adults by sex and age predict cardiometabolic risk factors in a cross-sectional study
Source: BMC Nutr. 2021 Jun 22;7:30. doi: 10.1186/s40795-021-00432-4 (PMC8218401; doi:10.1186/s40795-021-00432-4)
Supplement: Supplementary file 4 — Additional file 4: Supplemental Table 2. HEI-2015 in a cross-sectional study. Mean values of HEI-2015 components in women and men of three different ages categories in the WHNRC Nutritional Phenotyping Study. [file 40795_2021_432_MOESM4_ESM.docx]

| **Supplemental Table 2.** Mean values of HEI-2015 components in women and men of three different ages categories in the WHNRC Nutritional Phenotyping Study ^1^ | | | | | | | | | | |
| --- | --- | --- | --- | --- | --- | --- | --- | --- | --- | --- |
|  | Sex by age | | | | | | SEM | *P-value* | | |
|  | Women | | | Men | | |  |  |  |  |
| HEI-2015 | 18-33 y (n =73) | 34-49 y (n =67) | 50-65 y (n =66) | 18-33 y (n =60) | 34-49 y (n =59) | 50-65 y (n =53) |  | sex | age | sex  *age |
| Total-HEI | 60.4^c^ | 60.7^bc^ | 64.9^ab^ | 57.5^c^ | 61.0^bc^ | 66.3^a^ | 1.85 | 0.7 | **<0.01** | **<0.01** |
| Total Fruits | 2.86^a^ | 3.11^a^ | 3.12^a^ | 1.96^b^ | 2.88^a^ | 3.16^a^ | 0.25 | 0.07 | **0.01** | **<0.01** |
| Whole Fruits | 3.35^a^ | 3.75^a^ | 3.61^a^ | 2.40^b^ | 3.34^a^ | 3.77^a^ | 0.23 | **0.02** | **<0.01** | **<0.01** |
| Total Vegetables | 4.06 | 3.93 | 4.23 | 3.86 | 3.89 | 3.79 | 0.18 | **0.03** | 0.62 | 0.21 |
| Greens and Beans | 3.74 | 3.86 | 3.78 | 3.62 | 3.78 | 3.76 | 0.23 | 0.5 | 0.4 | 0.8 |
| Whole Grain | 3.48^ab^ | 2.77^a^ | 4.01^bc^ | 2.93^a^ | 2.98^a^ | 4.65^c^ | 0.37 | 0.9 | **<0.01** | **<0.01** |
| Dairy | 5.30 | 5.66 | 6.21 | 5.22 | 5.91 | 5.28 | 0.36 | 0.09 | 0.3 | 0.2 |
| Total Protein | 4.70 | 4.60 | 4.76 | 4.83 | 4.80 | 4.86 | 0.07 | 0.06 | 0.1 | 0.1 |
| Seafood and Plant Protein | 4.09 | 4.14 | 4.13 | 4.00 | 4.07 | 4.46 | 0.18 | 0.6 | 0.4 | 0.4 |
| Fatty Acids | 5.65 | 5.33 | 5.71 | 5.70 | 5.60 | 6.71 | 0.42 | 0.3 | 0.3 | 0.4 |
| Refined Grain | 7.00^a^ | 7.14^a^ | 8.43^b^ | 6.42^a^ | 7.24^a^ | 7.22^a^ | 0.36 | 0.1 | **0.01** | **0.02** |
| Sodium | 2.82^ab^ | 3.63^bc^ | 3.42^bc^ | 2.04^a^ | 3.39^b^ | 4.54^c^ | 0.39 | 1.0 | **<0.01** | **<0.01** |
| Added Sugar | 8.46^ab^ | 7.78^b^ | 8.60^a^ | 8.92^a^ | 8.30^ab^ | 8.67^a^ | 0.24 | 0.06 | **0.05** | **0.04** |
| Saturated Fats | 5.01^a^ | 4.95^a^ | 5.36^a^ | 5.56^a^ | 5.25^a^ | 6.89^b^ | 0.37 | **0.03** | 0.07 | **0.03** |
| ^1^Mean differences of untransformed HEI-components for sex, age and interactions were compared using Wilcoxon signed-rank tests for  non-parametric Wilcoxon-test. | | | | | | | | | | |
