# Supplementary material for: Healthy eating index patterns in adults by sex and age predict cardiometabolic risk factors in a cross-sectional study
Source: BMC Nutr. 2021 Jun 22;7:30. doi: 10.1186/s40795-021-00432-4 (PMC8218401; doi:10.1186/s40795-021-00432-4)
Supplement: Supplementary file 5 — Additional file 5: Supplemental Table 3. Correlations of HEI-components. Pearson correlation of the HEI-2015 components of the WHNRC Nutritional Phenotyping Study cohort. [file 40795_2021_432_MOESM5_ESM.docx]

**Supplemental Table 3** Pearson correlation of the HEI-2015 components of the WHNRC Nutritional Phenotyping Study cohort (n = 378)

|  | | | | | | | | | | | | | | | |
| --- | --- | --- | --- | --- | --- | --- | --- | --- | --- | --- | --- | --- | --- | --- | --- |
|  | Total Fruits | Whole Fruits | Total Vegetables | Greens and Beans | Whole Grain | Dairy | Total Protein | Seafood and Plant Protein | Fatty Acids | Refined Grain | Sodium | Added Sugar | Saturated Fats | Total score |  |
| Total Fruits | 1.00 |  |  |  |  |  |  |  |  |  |  |  |  |  |  |
| Whole Fruits | **0.87** | 1.00 |  |  |  |  |  |  |  |  |  |  |  |  |  |
| Total Vegetables | 0.24 | 0.29 | 1.00 |  |  |  |  |  |  |  |  |  |  |  |  |
| Greens and Beans | 0.19 | 0.24 | ***0.63*** | 1.00 |  |  |  |  |  |  |  |  |  |  |  |
| Whole Grain | 0.23 | 0.27 | 0.15 | 0.23 | 1.00 |  |  |  |  |  |  |  |  |  |  |
| Dairy | -0.13 | NS | NS | NS | NS | 1.00 |  |  |  |  |  |  |  |  |  |
| Total Protein | NS | NS | 0.21 | 0.32 | NS | NS | 1.00 |  |  |  |  |  |  |  |  |
| Seafood and Plant Protein | 0.20 | 0.23 | 0.37 | ***0.45*** | 0.27 | NS | ***0.40*** | 1.00 |  |  |  |  |  |  |  |
| Fatty Acids | 0.19 | 0.19 | 0.34 | 0.33 | 0.27 | ***-0.42*** | 0.21 | 0.32 | 1.00 |  |  |  |  |  |  |
| Refined Grain | 0.20 | 0.22 | 0.33 | 0.22 | 0.23 | NS | 0.31 | 0.38 | 0.24 | 1.00 |  |  |  |  |  |
| Sodium | 0.28 | 0.27 | -0.22 | -0.13 | 0.14 | NS | -0.14 | 0.12 | NS | NS | 1.00 |  |  |  |  |
| Added Sugar | NS | NS | ***0.43*** | 0.35 | 0.20 | NS | 0.31 | 0.26 | 0.22 | 0.22 | -0.31 | 1.00 |  |  |  |
| Saturated Fats | 0.35 | 0.29 | 0.21 | 0.21 | 0.34 | -0.36 | NS | 0.19 | **0.73** | NS | 0.10 | NS | 1.00 |  |  |
| Total Score | ***0.59*** | ***0.56*** | ***0.43*** | ***0.45*** | ***0.60*** | NS | 0.11 | ***0.49*** | ***0.61*** | ***0.53*** | 0.28 | 0.31 | ***0.62*** | 1.00 |  |
| The covariance matrix measures the degree to which a pair of variable change together (p<0.05).  Correlations of transformed HEI-components were considered **strong** (\|0.7\| ≥ r <\|1\|), ***moderate*** (\|0.4\| ≥ r <\|0.7\|) or weak (\|0.1\| ≥ r < 0.4)  No significant (NS) | | | | | | | | | | | | | | | |
