# Supplementary material for: Healthy eating index patterns in adults by sex and age predict cardiometabolic risk factors in a cross-sectional study
Source: BMC Nutr. 2021 Jun 22;7:30. doi: 10.1186/s40795-021-00432-4 (PMC8218401; doi:10.1186/s40795-021-00432-4)
Supplement: Supplementary file 6 — Additional file 6: Supplemental Table 4. Prevalence of metabolic variables in a cross-section study. Number of participants with high risk metabolic variable and their proportion (%) in the WHNRC Nutritional Phenotyping study high and low CVD-risk groups. [file 40795_2021_432_MOESM6_ESM.docx]

| **Supplemental Table 4**. Number of participants with high risk metabolic variable and their proportion (%) in the WHNRC Nutritional Phenotyping study high and low CVD-risk groups | | | | | |
| --- | --- | --- | --- | --- | --- |
| **High-risk group (n =296)** | 1-factor | 2-factors | 3-factors | 4-factors | 5-factors |
|  | 125(42) | 79(27) | 64(22) | 21(7) | 7(2) |
|  |  |  |  |  |  |
|  | BMI | HDLc | TG | HOMA | HbA1C |
| BMI | 248(84) |  |  |  |  |
| HDLc | 119(40) | 151(51) |  |  |  |
| TG | 37(13) | 32(11) | 40(14) |  |  |
| HOMA | 112 (38) | 80 (27) | 30(12) | 129(44) |  |
| HbA1c | 21(7) | 13(4) | 8(3) | 15(5) | 26(9) |
| **Low-risk group (n=97)** | | | |  |  |
|  | BMI | HDLc | TG | HOMA | HbA1C |
| BMI | 0 |  |  |  |  |
| HDLc | 0 | 0 |  |  |  |
| TG | 0 | 0 | 0 |  |  |
| HOMA | 0 | 0 | 0 | 0 |  |
| HbA1c | 0 | 0 | 0 | 0 | 0 |
| Study participants were classified *a priori* for a cardiometabolic outcome. High risk was based on at least one of the following criteria: BMI (kg/m^2^) of 25–44; fasting triglycerides >150 mg/dL; HDLc <50 mg/dL-women or <40 mg/dL-men; HOMA >2; HbA1c ≥5.7 and <6.5. Low risk was based on the absence of all risk factors. | | | | | |
