# Supplementary material for: Healthy eating index patterns in adults by sex and age predict cardiometabolic risk factors in a cross-sectional study
Source: BMC Nutr. 2021 Jun 22;7:30. doi: 10.1186/s40795-021-00432-4 (PMC8218401; doi:10.1186/s40795-021-00432-4)
Supplement: Supplementary file 7 — Additional file 7: Supplemental Table 5. Discriminant model of cardiometabolic risk. Performance of discriminant models of cardiometabolic risk groups using all HEI-2015 components. [file 40795_2021_432_MOESM7_ESM.docx]

**Supplemental Table 5**. Performance of discriminant models of cardiometabolic risk groups using all HEI-2015 components

|  | | | | | | |
| --- | --- | --- | --- | --- | --- | --- |
|  |  | **Percent Predicted (%)** | |  |  |  |
| **Age (y)** | **N** | **Low-risk**  **(n=97)** | **High-risk**  **(n=281)** | **AUC** | **Entropy R^2^** | **Prob>F** |
| ***Men & Women*** | | | | | | |
| 18 to 65* | 378 | 79 | 71 | 0.82 | 0.17 | <0.01 |
| 18 to 33 | 133 | 81 | 92 | 0.89 | 0.61 | 0.06 |
| 34 to 49 | 126 | 50 | 72 | 0.84 | 0.53 | 0.26 |
| 50 to 65 | 119 | 70 | 72 | 0.88 | 0.59 | 0.13 |
| ***Women*** | | | | | | |
| 18 to 65 | 206 | 68 | 82 | 0.86 | 0.35 | <0.01 |
| 18 to 33 | 73 | 60 | 72 | 0.91 | 0.82 | 0.25 |
| 34 to 49 | 67 | 70 | 77 | 0.93 | 0.54 | 0.15 |
| 50 to 65 | 66 | 100 | 100 | 0.91 | 0.97 | 0.33 |
| ***Men*** | | | | | | |
| 18 to 65 | 172 | 85 | 86 | 0.83 | 0.50 | 0.52 |
| 18 to 33 | 60 | 100 | 100 | 0.95 | 0.94 | 0.55 |
| 34 to 49 | 59 | 80 | 98 | 0.89 | 0.71 | 0.57 |
| 50 to 65 | 53 | 100 | 88 | 0.82 | 0.77 | 0.09 |
| *All HEI-components including sex and age as covariate in the model: low risk, 47%; high-risk 92%; AUC=0.82; entropy R^2^=0.15; *P* =0.04. | | | | | | |
