# Supplementary material for: Healthy eating index patterns in adults by sex and age predict cardiometabolic risk factors in a cross-sectional study
Source: BMC Nutr. 2021 Jun 22;7:30. doi: 10.1186/s40795-021-00432-4 (PMC8218401; doi:10.1186/s40795-021-00432-4)
Supplement: Supplementary file 8 — Additional file 8: Supplemental Table 6. Nominal logistic regression of cardiometabolic risk. Performance of nominal logistic regression of cardiometabolic risk groups using all HEI-2015 components. [file 40795_2021_432_MOESM8_ESM.docx]

**Supplemental Table 6**. Performance of nominal logistic regression of cardiometabolic risk groups using all HEI-2015 components

|  | | | | | | |
| --- | --- | --- | --- | --- | --- | --- |
|  |  | **Percent Predicted (%)** | |  |  |  |
| **Age (y)** | **N** | **Low-risk** | **High-risk** | **AUC** | **Entropy R^2^** | **Prob>F** |
| ***Men & Women*** | | | | | | |
| 18 to 65 | 378 | 93 | 98 | 0.70 | 0.07 | 0.04 |
| 18 to 33 | 133 | 41 | 90 | 0.80 | 0.20 | 0.04 |
| 34 to 49 | 137 | 81 | 98 | 0.79 | 0.16 | 0.23 |
| 50 to 65 | 107 | 63 | 95 | 0.80 | 0.22 | 0.11 |
| ***Women*** | | | | | | |
| 18 to 65 | 206 | 30 | 93 | 0.80 | 0.19 | <0.01 |
| 18 to 33 | 73 | 50 | 93 | 0.84 | 0.25 | 0.17 |
| 34 to 49 | 67 | 53 | 92 | 0.87 | 0.36 | 0.04 |
| 50 to 65 | 66 | 60 | 97 | 0.93 | 0.52 | 0.02 |
| ***Men*** | | | | | | |
| 18 to 65 | 172 | 7 | 97 | 0.73 | 0.11 | 0.31 |
| 18 to 33 | 60 | 100 | 100 | 0.91 | 0.42 | 0.08 |
| 34 to 49 | 59 | 72 | 100 | 0.93 | 0.71 | 0.10 |
| 50 to 65 | 53 | 60 | 100 | 0.87 | 0.34 | 0.28 |
|  |  |  |  |  |  |  |
