# Supplementary material for: Healthy eating index patterns in adults by sex and age predict cardiometabolic risk factors in a cross-sectional study
Source: BMC Nutr. 2021 Jun 22;7:30. doi: 10.1186/s40795-021-00432-4 (PMC8218401; doi:10.1186/s40795-021-00432-4)
Supplement: Supplementary file 9 — Additional file 9: Supplemental Table 7. Nominal logistic regression of cardiometabolic risk using selected HEI-2015 components. Performance of nominal logistic regression of cardiometabolic risk groups using selected HEI-2015 components. [file 40795_2021_432_MOESM9_ESM.docx]

| **Supplemental Table 7**. Performance of nominal logistic regression of cardiometabolic risk groups using selected HEI-2015 components | | | | | | |
| --- | --- | --- | --- | --- | --- | --- |
|  | | | | | | |
|  |  | **Percent Predicted (%)** | |  |  |  |
| **Age (y)** | **Model components** | **Low-risk** | **High-risk** | **AUC** | **Entropy R^2^** | **Prob >F** |
| ***Women*** | | | | | | |
| 18 to 33  (n =73) | Dairy, ToPro, ToVeg, rGr, FAs, Sod, SatFat, ToFru, rGr AdSug, | 31 | 90 | 0.78 | 0.19 | 0.08 |
| 34 to 49  (n = 67) | Dairy, wFru, ToPro, ToVeg, rGr, S&PPro, Sod, G&B, SatFat, ToFru, wGr, | 53 | 93 | 0.88 | 0.35 | 0.01 |
| 50 to 65  (n =66) | Dairy, wFru, ToPro, rGr, FAs, AdSu, Sod ToFru, , wGr, | 60 | 100 | 0.91 | 0.50 | 0.02 |
| ***Men*** | | | | | | |
| 18 to 33  (n = 60) | Dairy, wFruit, ToPro, ToVeg, rGr, FAs, S&PPro, AdSug, G&B, wGr | 63 | 92 | 0.91 | 0.41 | <0.01 |
| 34 to 49  (n = 59) | Dairy, wFru, ToPro, ToVeg, rGr, FAs, S&PPro, AdSug, G&B | 29 | 98 | 0.87 | 0.30 | 0.03 |
| 50 to 65  (n = 53) | Dairy, wFru, ToPro, ToVeg, FAs, S&Pro, Sod, G&B, SatFat | 33 | 94 | 0.77 | 0.18 | 0.31 |

HEI-components ranked by frequency of appearance in models: HEI-components ranked by frequency of appearance in models: Dairy (n =6); whole-fruit (wFru; n =5); total protein (ToPro; n =5); total vegetables (ToVeg; n =5); refined-grains (rGr; n =5); fatty acids (FAs; n =5); sea-food and plants (S&PPro; n =4); Added sugars (AdSugS; n =4); sodium (Sod; n =4); greens and beans (G&B; n =4); saturated fat (satFat; n =3); total-fruits (ToFru; n =3); whole-grain (wGr; n =3).
