# Supplementary material for: Healthy eating index patterns in adults by sex and age predict cardiometabolic risk factors in a cross-sectional study
Source: BMC Nutr. 2021 Jun 22;7:30. doi: 10.1186/s40795-021-00432-4 (PMC8218401; doi:10.1186/s40795-021-00432-4)
Supplement: Supplementary file 10 — Additional file 10: Supplemental Table 8. Stepwise discriminant analysis. HEI-2015 component selection in the WHNRC Nutritional Phenotyping study cohort by stepwise discriminant analysis. [file 40795_2021_432_MOESM10_ESM.docx]

**Supplemental Table 8.** HEI-2015 component selection in the WHNRC Nutritional Phenotyping study cohort by stepwise discriminant analysis

|  |  |  | *Women* | | | | | | | | | | | | |
| --- | --- | --- | --- | --- | --- | --- | --- | --- | --- | --- | --- | --- | --- | --- | --- |
| Age (y) | n | Step | 1 | 2 | 3 | 4 | 5 | 6 | 7 | 8 | 9 | 10 | 11 | 12 | 13 |
| 18-33 | 73 |  | **AdSu** | **ToVeg** | **Sod** | **Dairy** | **G&B** | **rGr** | **ToPro** | **SatFat** | **FAs** | **ToFru** | WGr | S&PPro | Wfru |
|  |  | R^2^ | 0.07 | 0.06 | 0.08 | 0.18 | 0.23 | 0.40 | 0.35 | 0.48 | 0.57 | **0.66** | 0.51 | 0.70 | 0.72 |
|  |  | -2LL | 74.7 | 71.3 | 66.7 | 56.8 | 52.3 | 42.3 | 45.0 | 36.5 | 29.9 | **23.8** | 33.7 | 20.9 | 19.1 |
| 34-49 | 67 |  | **WFru** | **SatFat** | **Sod** | **ToVeg** | **ToFru** | **S&PPro** | **ToPro** | **Dairy** | **wGr** | **G&B** | rGr | FAs | AdSu |
|  |  | R^2^ | 0.05 | 0.03 | 0.10 | 0.25 | 0.31 | 0.36 | 0.46 | 0.46 | 0.71 | **0.75** | 0.75 | 0.79 | 0.72 |
|  |  | -2LL | 68.8 | 63.2 | 58.7 | 48.3 | 45.2 | 41.7 | 35.0 | 35.5 | 18.4 | **12.1** | 11.1 | 16.3 | 18.1 |
| 50-65 | 66 |  | **Sod** | **Adsu** | **Dairy** | **ToPro** | **rGr** | **wGr** | **S&PPro** | **ToVeg** | **SatFat** | **FAs** | G&B | ToFru | wFru |
|  |  | R^2^ | 0.02 | 0.09 | 0.15 | 0.14 | 0.30 | 0.67 | 0.76 | 0.77 | 0.78 | **0.85** | 0.8 | 0.69 | 0.65 |
|  |  | -2LL | 48.3 | 43.4 | 40.7 | 41.3 | 33.4 | 15.8 | 11.3 | 4.60 | 10.77 | **1.6** | 6.37 | 14.9 | 16.8 |
|  |  |  | *Men* | | | | | | | | | | | | |
| 18-33 | 60 |  | **G&B** | **ToVeg** | **SatFat** | **WFru** | **rGr** | **S&PPro** | **ToPro** | **FAs** | **wGr** | **Dairy** | **ToFru** | Sod | AdSu |
|  |  | R^2^ | 0.04 | 0.09 | 0.20 | 0.34 | 0.45 | 0.47 | 0.50 | 0.57 | 0.66 | 0.77 | **0.86** | 0.80 | 0.89 |
|  |  | -2LL | 47.4 | 45.3 | 39.5 | 33.1 | 27.1 | 26.0 | 24.9 | 0.21 | 18.5 | 16.9 | **6.72** | 7.77 | 7.52 |
| 34-49 | 59 |  | **ToVeg** | **rGr** | **FAs** | **S&PPro** | **wFru** | **ToPro** | **G&B** | **AdSu** | **SatFat** | **Dairy** | ToFru | Sod | wGr |
|  |  | R^2^ | 0.04 | 0.04 | 0.03 | 0.26 | 0.45 | 0.42 | 0.53 | 0.67 | 0.71 | **0.85** | 0.74 | 0.82 | 0.74 |
|  |  | -2LL | 48.96 | 45.6 | 38.4 | 29.2 | 21.6 | 20.0 | 18.2 | 10.5 | 15.5 | **7.88** | 14.2 | 18.9 | 68.8 |
| 50-65 | 53 |  | **ToVeg** | **G&B** | **FAs** | **Dairy** | **rGr** | **ToFru** | **SatFA** | **wGr** | wFru | AdSu | Sod | S&PPro | ToPro |
|  |  | R^2^ | 0.04 | 0.17 | 0.31 | 0.30 | 0.45 | 0.54 | 0.59 | **0.95** | 0.77 | 0.70 | 0.70 | 0.60 | 0.58 |
|  |  | -2LL | 38.5 | 33.1 | 0.27 | 28.2 | 14.1 | 14.4 | 4.57 | **1.53** | 3.33 | 8.41 | 8.03 | 15.8 | 16.6 |
|  |  |  | *All Subjects* | | | | | | | | | | | | |
| 18-55 | 377 |  | ToVeg | Sod | Dairy | WFru | AdSu | S&PPro | FAs | ToPro | G&B | ToFru | SatFA | rGr | WGr |
|  |  | R^2^ | 0.01 | 0.02 | 0.02 | 0.02 | 0.03 | 0.03 | 0.03 | 0.04 | 0.04 | 0.04 | 0.08 | 0.10 | 0.17 |
|  |  | -2LL | 375 | 362 | 353 | 342 | 342 | 338 | 330 | 328 | 314 | 314 | 300 | 295 | 273 |
| The step is the selection sequence of HEI-component by gender and age category. The numbers in bold are the HEI-components that were included in the final model. The entropy r-square (R2) and -2logLikelihood (-2LL) correspond to models built with all variables up to the included step. HEI-components ranked by frequency of appearance in final models: HEI-components ranked by frequency of appearance in models: Dairy (n =6); total-vegetables (ToVeg; n =6); saturated fat (satFat; n =6); greens and beans (G&B; n =5); total protein (ToPro; n =5); refined-grains (rGr; n =5); fatty acids (FAs; n =5); whole-grain (wGr; n =4); total-fruit (ToFru; n =4); sea-food and plants (S&PPRO; n =4); whole-fruits (WFru; n =3); Added sugars (AdSu; n =3); sodium (Sod; n =3). | | | | | | | | | | | | | | | |
