# Supplementary material for: Healthy eating index patterns in adults by sex and age predict cardiometabolic risk factors in a cross-sectional study
Source: BMC Nutr. 2021 Jun 22;7:30. doi: 10.1186/s40795-021-00432-4 (PMC8218401; doi:10.1186/s40795-021-00432-4)
Supplement: Supplementary file 11 — Additional file 11: Supplemental Table 9. Comparison of actual vs predicted cardiometabolic risk in a phenotyping study. Stepwise discriminant analysis with the total population or a modeling using women and men by age category. [file 40795_2021_432_MOESM11_ESM.docx]

**Supplemental Table 9.** Comparison of actual vs predicted cardiometabolic risk in a phenotyping study using stepwise discriminant analysis with the total population or a modeling using women and men by age category.

|  | Total |  | Women | | |  | Men | | |
| --- | --- | --- | --- | --- | --- | --- | --- | --- | --- |
| Risk Category | 18-65y  (n =378) |  | 18-33 y (n=73) | 34-49 y (n=67) | 50-65 y (n=66) |  | 18-33 y (n=60) | 34-49 y (n=59) | 50-65 y (n=53) |
|  |  |  |  |  |  |  |  |  |  |
| Low-risk *_(Actual)_* | 106 |  | 17 | 18 | 17 |  | 20 | 19 | 15 |
| High-risk *_(Actual)_* | 272 |  | 56 | 49 | 49 |  | 40 | 40 | 38 |
| % High-risk *_(Actual)_* | 72% |  | 77% | 75% | 74% |  | 67% | 68% | 72% |
|  | ***Misclassification (n)*** | | | | | | | | |
| Low-risk | 71 |  | 5 | 2 | 1 |  | 3 | -5 | 0 |
| High-risk | -71 |  | -5 | -4 | -4 |  | -3 | 1 | 5 |
| Total | 142 |  | 10 | 6 | 5 |  | 6 | 6 | 5 |
|  | ***Misclassification Rate (%)*** | | | | | | | | |
| Total | 38% |  | 14% | 9% | 8% |  | 10% | 10% | 9% |
