# Supplementary material for: Healthy eating index patterns in adults by sex and age predict cardiometabolic risk factors in a cross-sectional study
Source: BMC Nutr. 2021 Jun 22;7:30. doi: 10.1186/s40795-021-00432-4 (PMC8218401; doi:10.1186/s40795-021-00432-4)
Supplement: Supplementary file 12 — Additional file 12: Supplemental Table 10. Evaluation of the predictive model in an independent study of overweight women. Predicted healthy eating index (HEI)-components of high- cardiometabolic risk comparing a cross-sectional and targeted overweight study in women. [file 40795_2021_432_MOESM12_ESM.docx]

| **Supplemental Table 10.** Healthy eating index (HEI)-components by predicted high- cardiometabolic risk* comparing a cross-sectional and targeted overweight study in women. | | | | | |
| --- | --- | --- | --- | --- | --- |
|  | | | | | |
| HEI-Components | Type of Study | |  | *P* | |
|  | Overweight to obese^α^  (n= 43) | Cross-sectional  (n=150) | SEM | age | Study |
| Total Fruits | 2.97 | 2.79 | 0.35 | 0.38 | 0.68 |
| Whole Fruits | 3.90 | 3.33 | 0.36 | 0.11 | 0.12 |
| Total Vegetables | 3.99 | 3.85 | 0.24 | 0.89 | 0.39 |
| Greens and beans | 3.58 | 3.62 | 0.35 | 0.66 | 0.46 |
| Whole grain | 3.24 | 3.12 | 0.53 | **0.01** | 0.55 |
| Dairy | 5.36 | 5.91 | 0.51 | 0.51 | 0.25 |
| Total protein | 4.51 | 4.71 | 0.12 | 0.90 | 0.20 |
| Seafood and plant protein | 3.82 | 3.94 | 0.29 | 0.12 | 0.44 |
| Fatty acids | 3.92 | 5.06 | 0.62 | 0.60 | **0.04** |
| Refined grain | 6.62 | 7.05 | 0.57 | **<0.01** | 0.21 |
| Sodium | 3.58 | 3.04 | 0.55 | 0.07 | 0.16 |
| Added sugar | 7.31 | 8.01 | 0.40 | 0.10 | 0.24 |
| Saturated Fats | 4.13 | 4.63 | 0.61 | 0.62 | 0.44 |
| HEI-Total | 56.7 | 59.1 | 1.35 | **0.01** | 0.38 |
| *participants of the phenotyping study were classified for a cardiometabolic outcome based on at least one of the following criteria: BMI (kg/m2) of 25–44, HOMA>2, fasting triglyceride concentrations >150 mg/dL or HDL cholesterol <50 mg/dL  α The study of Individual Metabolism and Physiological Signatures (iMAPs; ClinicalTrials.gov: NCT02298725) was conducted in pre- and postmenopausal overweight to obese women (n =44) who had <150 min/wk of physical activity and ≥1 cardiometabolic risk factor. Inclusion criteria included age 20–65 y, BMI of 25–39.9 kg/m², and resting blood pressure ≤140/90 mm Hg, and evidence of impaired glucose homeostasis, elevated fasting triglycerides, and/or low HDL cholesterol (HDLc), as previously described. | | | | | |
