# Supplementary material for: Healthy eating index patterns in adults by sex and age predict cardiometabolic risk factors in a cross-sectional study
Source: BMC Nutr. 2021 Jun 22;7:30. doi: 10.1186/s40795-021-00432-4 (PMC8218401; doi:10.1186/s40795-021-00432-4)
Supplement: Supplementary file 13 — Additional file 13: Supplemental Table 11. Comparison of actual vs predicted HEI-components. HEI-components by predicted stepwise discriminative cardiometabolic risk in a cross-sectional study. [file 40795_2021_432_MOESM13_ESM.docx]

| **Supplemental Table 11.** HEI-components by predicted stepwise discriminative cardiometabolic risk in a cross-sectional study. | | | | | | | |
| --- | --- | --- | --- | --- | --- | --- | --- |
|  | | | | | | | |
| HEI-Components | Sex | Phenotyping study | |  | *P* | | |
|  |  | no-risk  (n= 97) | Risk  (n=280) | SEM | Risk*sex | Risk |  |
| Total Fruits | women | 3.68^a^ | 2.72^b^ | 0.19 | 0.01 | <0.01 |  |
|  | men | 2.60^b^ | 2.56^b^ | 0.17 |  |  |  |
| Whole Fruits | women | 4.29^a^ | 3.05^b^ | 0.21 | 0.01 | <0.01 |  |
|  | men | 3.25^b^ | 3.05^b^ | 0.23 |  |  |  |
| Total Vegetables | women | 4.59 | 3.89 | 0.18 | 0.79 | <0.01 |  |
|  | men | 4.33 | 3.66 | 0.15 |  |  |  |
| Greens and beans | women | 4.27 | 3.62 | 0.18 | 0.54 | <0.01 |  |
|  | men | 4.43 | 3.47 | 0.16 |  |  |  |
| Whole grain | women | 3.90^ab^ | 3.01^b^ | 0.33 | 0.01 | <0.01 |  |
|  | men | 4.27^b^ | 2.89^a^ | 0.35 |  |  |  |
| Dairy | women | 4.35^a^ | 5.63^b^ | 0.32 | 0.01 | 0.05 |  |
|  | men | 5.25^ab^ | 5.94^b^ | 0.31 |  |  |  |
| Total protein | women | 4.61 | 4.71 | 0.07 | 0.10 | 0.68 |  |
|  | men | 4.85 | 4.81 | 0.06 |  |  |  |
| Seafood and plant protein | women | 4.46^a^ | 3.88^b^ | 0.15 | 0.02 | 0.07 |  |
|  | men | 4.17^ab^ | 4.17^ab^ | 0.17 |  |  |  |
| Fatty acids | women | 6.48^ab^ | 4.90^c^ | 0.32 | 0.01 | <0.01 |  |
|  | men | 6.94^a^ | 5.31^bc^ | 0.35 |  |  |  |
| Refined grain | women | 8.59^a^ | 7.13^b^ | 0.38 | 0.05 | 0.03 |  |
|  | men | 6.95^b^ | 6.88^b^ | 0.34 |  |  |  |
| Sodium | women | 4.11^a^ | 2.68^b^ | 0.30 | 0.05 | 0.18 |  |
|  | men | 3.14^sb^ | 3.33^sb^ | 0.28 |  |  |  |
| Added sugar | women | 8.51^b^ | 8.04^b^ | 0.18 | 0.01 | <0.01 |  |
|  | men | 9.42^a^ | 8.12^b^ | 0.20 |  |  |  |
| Saturated Fats | women | 5.91^a^ | 4.52^b^ | 0.33 | 0.05 | <0.01 |  |
|  | men | 6.29^a^ | 5.51^ab^ | 0.40 |  |  |  |
| HEI-Total | women | 68.3 | 57.5 | 1.23 | 0.26 | <0.01 |  |
|  | men | 65.7 | 58.8 | 1.06 |  |  | |
